# The Use of LPC and Wavelet Transform for Influenza Disease Modeling

**DOI:** 10.3390/e20080590

**Published:** 2018-08-09

**Authors:** Khaled Daqrouq, Mohammed Ajour

**Affiliations:** Department of Electrical and Computer Engineering, King Abdulaziz University, P.O. Box 80204, Jeddah 21589, Saudi Arabia

**Keywords:** LPC, wavelet transform, speech, influenza disease modeling

## Abstract

In this paper, we investigated the modeling of the pathological features of the influenza disease on the human speech. The presented work is novel research based on a real database and a new combination of previously used methods, discrete wavelet transform (DWT) and linear prediction coding (LPC). Three verification system experiments, Normal/Influenza, Smokers/Influenza, and Normal/Smokers, were studied. For testing the proposed pathological system, several classification scores were calculated for the recorded database, from which we can see that the proposed method achieved very high scores, particularly for the Normal with Influenza verification system. The performance of the proposed system was also compared with other published recognition systems. The experiments of these schemes show that the proposed method is superior.

## 1. Introduction

As humans, we interact with each other in many ways, such as talking, writing, by signs, and so on, the most commonly used of which is talking. Human speech is a sound; the sound is a mechanical wave of pressure and displacement. The human speech is formed with the help of many organs, such as the lungs, larynx, throat, nose, mouth, sinuses, and other secondary organs. The air exhaled through these organs, which comes from the lungs through the vocal folds inside the larynx, provides energy for the vocal folds to produce the sound. The other organs then help to formulate the sound into speech [1,2,3,4].

Pathology is the science of causes and effects of diseases. The development of this field depends on the development of other related sciences, and technology will produce new diagnosing methods and more accurate disease diagnoses [5,6,7,8,9,10]. The human vocal tract, as with any human organ, is affected by many factors, including aging, diseases, or congenital flaw. These factors affect their functionality with different levels of effects, and somehow change the sound these organs produce. Every pathological (disease) factor may have specific changes in the human sound, modeling these changes will enable us to detect some diseases by the sound of the patient [11,12,13,14].

Many types of research have been applied in voice pathology diagnostics, such as characterization methods for the detection of multiple voice disorders: neurological, functional, and laryngeal diseases. In this research, many methods are tested, such as a Mel-frequency cepstral coefficient (MFCC) and a Gaussian classifier for modeling the pathology by speech. The study came up with a range of accuracies between 81% and 98% for different pathologies. Pathological voice recognition for vocal fold disease was investigated by Gaussian mixture model [14], where a range of accuracies between 83% and 98% were achieved.

Researchers have used different ideas to achieve better performance of feature extraction methods. In Tohan et al. [15], acoustic parameters using multi corpus optimization with a neural network based support vector machine (SVM) classifier was proposed. Linear prediction coding (LPC), LPCC, MFCC, and PLP were used by Palo et al. [16] and Selvasraj et al. [17], and modified by Pao et al. [18] and Sato et al. [19]. Feature extraction methods based on wavelet transform were proposed in many works [20,21,22]. Wang et al. [23] investigated the use of a Fourier-based method for better feature extraction quality.

In this paper, wavelet transform is used in conjunction with LPC for speech pathology modeling and recognition. For classification, probabilistic neural network (PNN) is used. The verification task is conducted for investigation of the method quality.

## 2. The Method

In this paper, we investigated the pathological modeling of the influenza disease. Novel research based on a real database and a new combination of previously used methods, wavelet transform and LPC, was proposed. Wavelet transform is a very attractive approach for many researchers in the pattern recognition field and signal processing [13]. The main reason behind this is the possibility to decompose the signal into sub-signals of different bandpasses of frequency. The reason behind using the discrete wavelet transform (DWT) method is the advantage of decomposing the signal into different pass bands of frequency for each sub-signal. Thus, there is no frequency overlapping. It is highly effective in achieving a better feature extraction possibility. By utilizing the LPC for each sub-signal to be linked into one vector containing all LPC coefficients of these sub-signals, we get a feature extraction vector. The level of DWT was chosen empirically for the best performance. The determination of DWT of level 5 was adopted as a tradeoff between better performance and less dimensionality of the feature vector [24].

In this paper, we used the following steps:

• Decomposing the speech signal into DWT of level 5:

(1) $$F=\left[D_{1},\;D_{2},\;D_{3},D_{4},\;D_{5},A_{5}\right]$$

where,

(2) $$\begin{array}{c} D_{1}=d_{11},d_{12},\ldots ,d_{1N/2}\\ D_{2}=d_{21},d_{22},\ldots ,d_{2N/4}\\ D_{4}=d_{31},d_{32},\ldots ,d_{3N/8}\\ D_{4}=d_{41},d_{42},\ldots ,d_{4N/16}\\ D_{5}=d_{51},d_{52},\ldots ,d_{5N/32}\\ A_{5}=d_{51},d_{52},\ldots ,d_{5N/32} \end{array}$$

where $D_{1}$ is the detail DWT sub-signal of level l that represented the high frequency part of the original signal x(t), and was calculated as a convolution of the signal with the basis $\psi_{1,k}\left(t\right)$ generated from the mother wavelet function. For the DWT of level *j* = 1:

(3) $$D_{1}=x\left(t\right)*\psi_{1,k}\left(t\right)$$

where,

(4) $$\psi_{1,k}\left(t\right)=2^{-\frac{1}{2}}\psi \left(2^{-1}t-k\right)$$

and *k* = 1, 2, …, *N*/2; *N* is the number of the data record; and $A_{J}$ is the approximated DWT sub-signal of the last level *L* that represented the low frequency part of the original signal x(t), and was calculated as a convolution of the signal with the basis $\varphi_{J,k}\left(t\right)$ generated from the father wavelet function [13]:

(5) $$A_{J}=x\left(t\right)*\varphi_{J,k}\left(t\right)$$

For the DWT of level *j* = *J*:

(6) $$\psi_{J,k}\left(t\right)=2^{-\frac{J}{2}}\varphi \left(2^{-J}t-k\right)$$

• Calculating LPC of each sub-signal of 30 coefficients as follows:

(7) $$lpc_{l}=\mathrm{LPC}\left(d_{l}\right)$$

where *lpc* calculated the coefficients of a forward linear predictor by minimizing the prediction error in the least squares sense. The *lpc* was applied in the speech coding and filter design. The Matlab function LPC found the coefficients of a pth-order linear predictor. LPC predicted the present value based on the past samples as follows: −*a*(2) × (*n* − 1)−*a*(3) × (*n* − 2)−…−*a*(*p* + 1) × (*n* − *p*), where *p* is the order and a is [1 *a*(2) ... *a*(*p* + 1)]. The determination of the number of LPC coefficients (exactly as in the DWT level) was adopted as a tradeoff between better performance and less dimensionality of the feature vector [13].

• The feature extraction vector is

(8) $$FV=\left[lpc_{1},lpc_{2},lpc_{3},lpc_{4},lpc_{5},lpc_{a5}\right]$$

The choice of the wavelet mother function type is crucial and is dependent on the intended application. In our study, we investigated many wavelet functions and their corresponding recognition rate. Based on our investigation, we chose to use the wavelet function type Daubechies five (also known as db5) on the basis that it yielded the best recognition rate. Therefore, it was considered for rest of our investigation.

For classification, a probabilistic neural network was proposed. Ganchev proposed PNN with Mel-frequency cepstral coefficients for classification [25]. Although, there does exist numerous modified versions of the original PNN that are more economical and offer better performance. For simplicity of the exposition, we implemented the original PNN for the classification task. The proposed algorithm was of the following construction:

Net = PNN (X, R, SP)(9)

where X is the matrix of 180X8 input speech feature vectors (pattern) of 180 LPC coefficients; R is the target class vector, R = [1, 2, 3, …, 8]; and SP is the spread. The number 180 came from the idea that we calculated six DWT sub-signals: D1, D2, D3, D4, D5, and A5. Then, we calculated 30 LPC coefficients for each sub-signal. After that, they were linked together in one feature vector of 180 coefficients in length. The number, “eight”, came from the training signal, “four”, that was used for the training of each type at each verification system. Four signals of each type were recorded separately for training tasks along with the recorded testing database. For example, for classification of normal/influenza, four of each type were trained. If the system recognized the testing signal as one of the first four that belonged to normal cases, it was detected as the normal type, but if it recognized the testing signal as one of the second four, it was detected as the influenza type.

## 3. Results and Discussion

For testing of the proposed system, a suitable data set was recorded. For this purpose, 101 persons were involved in the dataset recording. Each participant had to record his speech with the same microphone in a quiet university room. The sampling frequency was 8 KHz. Twenty-two of the total people engaged in the recording had influenza disease in different levels, 48 smoking persons, and 41 normal persons, without influenza disease (normal). All participants were males aged between 20 and 27 years old. Four additional signals for each type were recorded for different participants. The four speech signals were utilized for the training process; therefore, all other recorded signals were used as testing signals. One Arabic sentence was recorded by all participants, which was composed of four words, and means, “peace, mercy, and blessings of God”.

The authors modeled all signals, the training and testing signals, by wavelet and LPC, and then trained the four additional speech signals for each type by PNN in Matlab. The models of testing signals were compared with the trained models by the simulation process offered in Matlab using the “sim” function. The verification system was adopted in the proposed study to investigate the accuracy of the presented approach, which used two types of the speech signal in the system; for example, Influenza/Normal. Influenza was determined as a positive class, and normal was determined as a negative class. Moreover, four parameters were crucial for the analysis of the results. These parameters were true positive (TP), false positive (FP), true negative (TN), and false negative (FN). The parameters mentioned were used to calculate several important specifications that are mentioned below.

**Figure 1:**
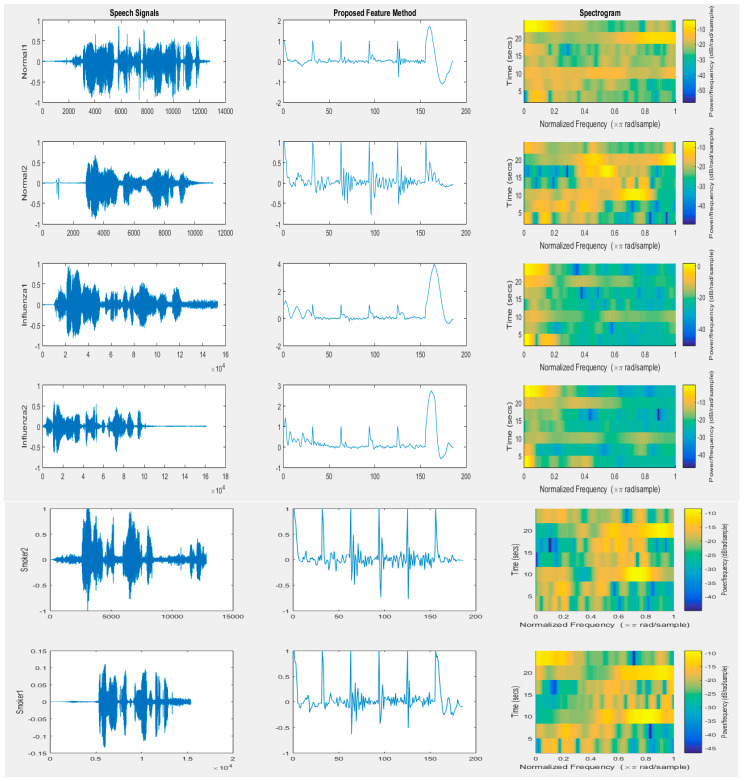
The speech signal for two normal people, two people with influenza disease, and two smokers; the feature vectors by the proposed method; and the spectrograms calculated for the feature vectors.

Figure 1 presents the speech signal for two normal people, two people with influenza disease, and two smokers. The feature vectors by the proposed method and spectrograms calculated for the feature vectors also are illustrated. By observing the feature vectors, as well as the spectrogram, we can easy recognize the normal case and the influenza case, as well as the smoker case, using these methods.

The recognition rate was calculated as an average of three measures:

(10) $$\begin{array}{c} (1)\;\mathrm{Sensitivity}\;\left(S\right)=\frac{\mathrm{TP}}{\mathrm{TP}+\mathrm{FN}}\times 100\%\\ (2)\;\mathrm{specificity}\;\left(P\right)=\frac{\mathrm{TN}}{\mathrm{TN}+\mathrm{FP}}\times 100\%\\ (3)\;\mathrm{positive}\;\mathrm{predictivity}\;\left(\mathrm{PP}\right)=\frac{\mathrm{TP}}{\mathrm{TP}+\mathrm{FP}}\times 100 \end{array}$$

In Table 1, three experiments are studied, namely the Normal/Influenza, Smokers/Influenza, and Normal/Smokers verification systems. The recognition rate was calculated as an average of the three measures S, P, and PP for each verification system.

Where TN is a true negative results number when the system identifies the testing signal as the normal case. TP is a true positive results number when the system confirms the tested signal as the influenza case, and in the case of the tested signal, really belongs to the influenza case. In other words, the criteria for determining the TP, TN, FN, and FP was based on categorization of the result of the testing samples into these four categories, such that if the tested sample was originally an influenza type for the Normal/Influenza verification system and the classifier recognized it as an influenza type, it was categorized as a true positive. However, if the classifier mistakenly recognized it as normal, it was categorized as a false negative.

If the tested sample was originally a normal type and the classifier recognized it as a normal type, it was categorized as a true negative. However, if the classifier mistakenly recognized it as an influenza type, it was categorized as a false positive. Then, the TP, TN, FN, and FP were the number of these cases for each category.

Other measures were also calculated for testing the method, such as the following measures:

- False rejection error (FRR), which is based on the false negative (FN) and true positive (TP) parameters as follows:
  (11) $$\mathrm{FRR}=\frac{\mathrm{FN}}{\left(\mathrm{TP}+\mathrm{FN}\right)}$$
- False acceptance error (FAR), which is based on false positive (FP) and true negative (TN) parameters as follows:
  (12) $$\mathrm{FAR}=\frac{\mathrm{FP}}{\left(\mathrm{FP}+\mathrm{TN}\right)}$$
- Accuracy (AC)
  (13) $$\mathrm{AC}=\frac{\mathrm{TP}+\mathrm{TN}}{\left(\mathrm{TP}+\mathrm{FN}+\mathrm{FP}+\mathrm{TN}\right)}$$
- Efficiency (EF)
  (14) $$\mathrm{EF}=100*\left[1-\frac{\mathrm{FN}}{\left(\mathrm{TP}+\mathrm{FN}\right)}\right]$$

For testing the proposed pathological system, all of the above classification scores were calculated for the recorded database for different Negative/Positive systems. The FAR, FRR, S, P, PP, AC, and EF scores were calculated (see Table 2), and we can see that the proposed method achieved very high scores, particularly for Normal with Influenza.

The value based on the recognition rate of the proposed systems was also compared with other published recognition systems, such as power spectrum density (PSD) [12], LPC [13], and MFCC [12]. Table 3 tabulates the comparative results obtained on the recorded database. The experiments of these schemes show that the proposed method is superior.

## 4. Conclusions

In this paper, we investigate the pathological modeling of the influenza disease using speech signal via testing over a recorded database. Novel research, based on original samples recorded in university circumstances, was suggested. A new combination of previously used methods, discrete wavelet transform and linear prediction coding (LPC), was proposed. Based on our investigation, we have chosen to use the wavelet function type Daubechies five (also known as db5) on the basis that it yields the best recognition rate. Three verification systems experiments, Normal/Influenza, Smokers/Influenza, and Normal/Smokers were studied. Many statistical scores were utilized for testing, such as sensitivity, specificity, false acceptance rate, false rejection rate, or efficiency. The performance of the proposed system was also compared with other published recognition systems. We can state that the proposed method achieved very high scores, particularly for the Normal with Influenza verification system.

## Figures and Tables

**Table 1:** Results of recognition rate for proposed method. TN—true negative; TP—true positive; FN—false negative; FN—false negative.

Negative/Positive	TN/TP	FN/FP	Recognition Rate
**Normal/Influenza**	37/22	0/4	92.60%
**Smokers/Influenza**	36/22	0/13	78.78%
**Normal/Smokers**	36/38	10/5	85.11%

**Table 2:** The results of different recognition statistical parameters for proposed method. FAR—false acceptance error; FRR—false rejection error; S—sensitivity; P—specificity; PP—positive predictivity; AC—accuracy; EF—efficiency.

Negative/Positive	FAR	FRR	S	P	PP	AC	EF
Normal/Influenza	9.76	0	100	90.62	84.24	93.65	100
Influenza/Smokers	26.53	0	100	73.47	62.86	81.69	100
Normal/Smokers	12.20	20.83	79.17	87.80	88.37	83.15	79.16

**Table 3:** Results of recognition rate for a different method for comparison. LPC—linear prediction coding; MFCC—Mel-frequency cepstral coefficient.

Normal/Influenza	TN/TP	FN/FP	Recognition Rate
**PSD**	33/18	4/8	77.18%
**LPC**	33/12	10/8	64.85%
**MFCC**	36/17	5/5	81.00%
**Proposed Method**	37/22	0/4	92.60%
